# Is a Ureteral Access Sheath Necessary for Maintaining Safe Intrarenal Pressures During Retrograde Lithotripsy Using a Flexible 7.5 Fr Scope and a High-Power TFL? In Vivo Experimental Study †

**DOI:** 10.3390/medicina61101829

**Published:** 2025-10-13

**Authors:** Athanasios Vagionis, Vasileios Tatanis, Angelis Peteinaris, Paraskevi Katsakiori, Vasiliki Tsekoura, Konstantinos Pagonis, Theofanis Vrettos, Evangelos Liatsikos, Panagiotis Kallidonis

**Affiliations:** 1 Department of Urology, University of Patras, 26504 Patras, Greece; thanos_vagionis@hotmail.gr (A.V.); peteinarisaggelis@gmail.com (A.P.); vkatsak@gmail.com (P.K.); vicktsek@yahoo.gr (V.T.); pagonisk7@gmail.com (K.P.); liatsikos@yahoo.com (E.L.); pkallidonis@yahoo.com (P.K.); 2 Department of Anesthesiology and ICU, University of Patras, 26504 Patras, Greece; teovret@gmail.com; 3 Department of Urology, Medical University of Vienna, 1090 Vienna, Austria

**Keywords:** intrarenal pressure, flexible ureteroscope, ureteral access sheath

## Abstract

*Background and Objectives*: To evaluate the effect of a ureteral access sheath (UAS) on the maximal intra-pelvic pressure (IPP max) during retrograde lithotripsy of hard and soft stones in a porcine model. *Materials and Methods*: A 22 Fr percutaneous tract was established in the upper calyces of the kidneys in three female pigs. A custom-made Foley catheter with a urodynamic catheter was inserted into the pelvicalyceal system and connected to a urodynamic device for real-time IPP measurement. A Pusen Uscope 7.5 Fr single-use ureteroscope (Zhuhai Pusen Medical Technology, Jinhua, China) with manual pump irrigation was used. BegoStone™ powder (Bego, Lincoln, RI, USA) was prepared in two powder-to-water ratios (15:3 and 15:6) to create hard and soft stones, respectively. Stones were positioned in the pelvicalyceal system through the percutaneous tract, and retrograde intrarenal lithotripsy was performed in three settings: without UAS and with a 9.5/11 Fr UAS, with lasing in the center of the pelvis, and during lithotripsy of soft and hard stones. *Results*: With manual pump irrigation and without a UAS, the IPP max reached 55 cmH_2_O during lasing in the pelvis center. During lithotripsy of soft and hard stones, the IPP max increased to 62 and 65 cmH_2_O, respectively. Using a UAS, the IPP max was significantly lower: 18 cmH_2_O in the center of the pelvis, and 25 and 29 cmH_2_O during lithotripsy of soft and hard stones, respectively. *Conclusions*: Manual pump irrigation without a UAS can elevate IPP max to potentially unsafe levels during retrograde correct flexible lithotripsy, even when using a 7.5 Fr flexible scope. The addition of a UAS helps maintain the IPP max within safer limits.

## 1. Introduction

Retrograde intrarenal surgery (RIRS) constitutes the gold standard procedure for treating ureteral and renal stones with diameters up to 2 cm in the renal pelvis and up to 1.5 cm in the lower pole according to the EAU guidelines [1]. Fluid irrigation is crucial to secure enough working space. Adequate fluid inflow is important to maintain safe intrarenal temperatures during laser lithotripsy. However, in the application of a setting without effective outflow, the intrarenal pressure increases to unsafe levels, which leads to the pyelotubular and pyelovenous backflow phenomena and is associated with complications such as postoperative fever and urosepsis [2,3,4]. Using large ureteral access sheaths improves the irrigation water outflow and maintains the intrarenal pressures at safe levels [5]. However, using large-diameter ureteral access sheaths in non-pre-stented ureters and ureters with strictures is often challenging and could lead to intraoperative and postoperative complications [6].

The introduction of newer single-use, smaller-diameter ureteroscopes offers the advantages of better intrarenal maneuverability and access to calyces with narrow infundibula [7]. Furthermore, they fit in smaller diameter access sheaths to perform an RIRS. As it has been previously described by Fang et al. [8] in an in vitro study, keeping the endoscope-sheath ratio to 0.75 or lower offers low intrapelvic pressures and acceptable irrigation flows. Thus, using smaller ureteroscopes allows using narrower sheaths while keeping the endoscope-sheath ratio to acceptable levels.

The purpose of the study is to evaluate the effect of using a 9.5–11.5 Fr ureteral access sheath on the maximal intra-pelvic pressure (IPP max) during retrograde lithotripsy of hard and soft stones with a high-power Thulium Fiber Laser (TFL) in an in vivo experimental setting.

## 2. Materials and Methods

### 2.1. Study Design and Experimental Setting

**Figure 1:**
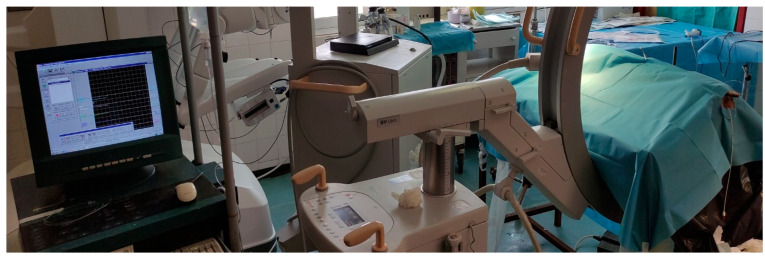
The use of the urodynamic system for the real-time measurement of the intrarenal pressure.

To perform this experimental in vivo study in porcine models, approval from the Veterinary State Services and the Hospital Ethics Committee was granted. Three porcine models were chosen for this pilot experimental study. The chosen sample size was chosen after considering ethical and practical considerations. As a scientific group, Russell’s 3R principle is always followed [9]; 3R stands for “Replacement”, “Reduction”, and “Refinement”. According to the “Reduction” principle, only enough animals should be used to assess feasibility while precluding needless suffering. In this study, efforts were made to obtain the greatest amount of data from the fewest animals possible. Notably, 6 kidneys of 3 pigs were tested, and each one of them was tested 3 times, providing us with data from 18 different cases. The chosen porcine models were all female crossbreeds from the Danish Landrace and American Duroc species. The porcine models were chosen due to the urological similarity of the porcine and the human kidneys [10]. Each animal weighed around 35 kilos (range, 30–40). An experimental setup was designed to measure real-time intrarenal pressure while performing RIRS in hard and soft stones with and without the use of an access sheath. Using an Amplatz renal dilator set (COOK Medical, Cook Ireland Ltd., Limerick, Ireland) to create a 22 Fr percutaneous tract, a urodynamic catheter connected to a urodynamic system was inserted through the upper calyces in the pelvicalyceal system of the three female porcine models. Percutaneous tract was established using fluoroscopy, the bullseye technique for the percutaneous puncture, and a one-step dilation technique up to 22 Fr in a similar manner with the mini percutaneous tract that has been described by the same scientific group [11]. The urodynamic system could measure and record in real-time the intrarenal pressure. A ureteral access sheath 9.5–11.5 Fr (Flexor^®^ Ureteral Access Sheath with AQR Hydrophilic Coating, COOK Medical, Cook Ireland Ltd., Limerick, Ireland) was inserted using fluoroscopy through the ureter with the tip just over the ureteropelvic junction. A 7.5 Fr novel single-use ureteroscope, Pusen Uscope 7.5 Fr PU3033A (Zhuhai Pusen Medical Technology, China), paired with manual pump irrigation, was used for the stone treatment. Gravity irrigation setting was excluded from this experiment as it is known that pairing gravity irrigation and high-power lithotripsy increases fluid temperature to unsafe levels [12]. Pusen scope offers a 650 mm working length, a 270° bending angle, a 3.6 working channel, and a 3 o’clock exit point of view [13]. The same procedure was performed in both kidneys of each animal, resulting in the collection of data from six different kidneys (Figure 1).

### 2.2. Preparation of the Porcine Models

The husbandry conditions followed the veterinary guidelines. To initiate the anesthesia part, a combination of three substances, including ketamine, xylazine, and atropine sulfate, was used. Each animal was set in a prone position. The ear veins of each porcine model were used to secure intravenous access. Subsequently, an endotracheal tube was placed and attached to mechanical ventilation. A 5% propofol solution was used to maintain anesthesia. Analytical information about the food and anesthesia of the animals has been extensively described in similar studies of our scientific group [14].

### 2.3. Operating Room Setup and Technique

**Figure 2:**
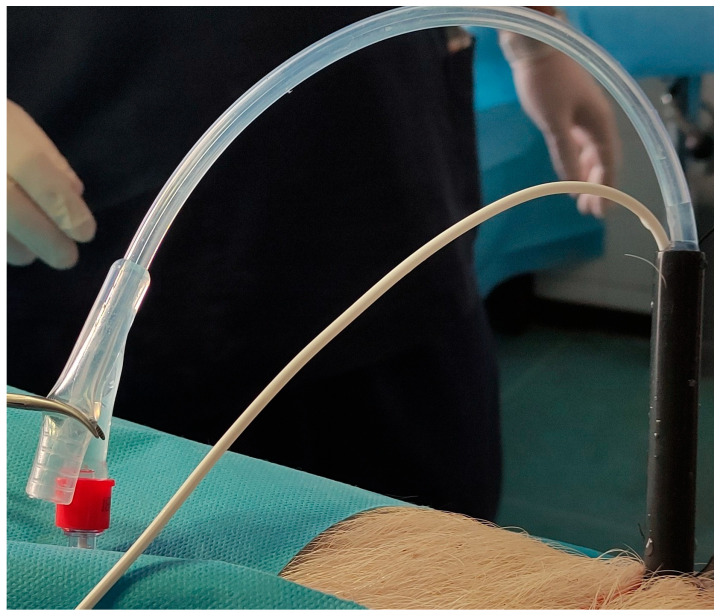
The custom-made balloon foley catheter.

**Figure 3:**
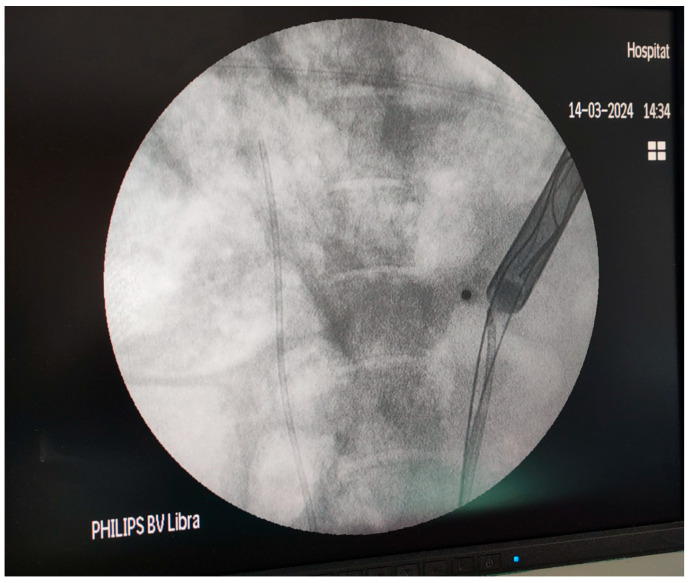
Experimental Setup.

Firstly, the pig was placed in the supine position. Using a mini-nephroscope, the ureteral orifices were located. A fully hydrophilic guidewire (HiWire^TM^ Nitinol Core Wire Guide, COOK Medical, Cook Ireland Ltd., Limerick, Ireland) was passed through the orifice to the pelvicalyceal system (PCS), and a 6 Fr open-end ureteral catheter (COOK Medical, Cook Ireland Ltd., Limerick, Ireland) was advanced over the guidewire to the renal pelvis of each kidney. Then the pig was turned to a prone position to perform the percutaneous access. A contrast medium was injected in the PCS, and a percutaneous puncture was performed in the upper calyces of the porcine kidneys using an open-end diamond tip needle (EchoTip^®^ needle, COOK Medical, Cook Ireland Ltd., Limerick, Ireland). An Amplatz renal dilator set (COOK Medical, Cook Ireland Ltd., Limerick, Ireland) was used to create a 22 Fr percutaneous tract. BegoStone™ powder (Begostone, Bego, Lincoln, RI, USA) and water were mixed in 15:3 and 15:6 powder-to-water ratios. Stones with 15:3 were defined as hard stones, while stones with 15:6 were defined as soft stones [15]. The utilized Begostone^TM^ mixtures were selected based on their similarity with human stones [16]. The mixture was kept for 72 h in a plastic mold. One hour prior to the experiment, the artificial stones were moistened in a water tank. A urodynamic catheter was inserted through the percutaneous tract in the pelvicalyceal system, and a custom-made balloon Foley catheter was used to stabilize the catheter and to seal the available space in the percutaneous tract. The balloon of the Foley catheter was inflated gradually to ensure that the percutaneous tract was air-tight and there was no pressure leak that could affect the measurements. The urodynamic catheter was carefully protected from the constriction by the possible excessive pressure of the balloon (Figure 2). The urodynamic catheter was connected with a Medtronic Dantec Duet Multi-P Urodynamic device (Medtronic Functional Diagnostics A/S, Copenhagen, Denmark) for real-time pressure measurement. Before every group of measurements, the urodynamic device was tested by flushing through the urodynamic catheter and then balanced to ensure that the urodynamic catheter was not affected by the balloon pressure. Hard and soft artificial stones were placed in the PCS using percutaneous access. Retrograde intrarenal lithotripsy was performed using a high-power Thulium Fiber Laser (TFL) (LaserClast Thulium Power^®^, EMS, Nyon, Switzerland) (Figure 3).

### 2.4. Measurement of the Intrarenal Pressure

Using a manual pump for irrigation, intrarenal pressure was measured. For each measurement, the TFL was activated in the setting of 1 J–60 Hz, resulting in a power level of 60 Watts (W), until a cumulative amount of energy of 3 KJ was emitted. The experiment was divided into two settings. Activating the laser in the center of the pelvis and urinary stone treatment. The stone treatment section included performing lithotripsy of hard and soft stones. Each experimental setting was tested with and without the use of a UAS. The experiment test was always performed in the same sequence, and a randomization protocol was not followed.

### 2.5. Statistical Analysis

Data calculations of the study were made with the IBM SPSS version 25 (IBM Corp., Armonk, NY, USA). Parameters were presented as mean value ± standard deviation. For the statistical analysis and *p*-value calculation, the Wilcoxon test was used. Statistical significance value was set as *p*-value < 0.05.

## 3. Results

Maximal intrapelvic pressures (IPPmax) mean values and their standard deviation are presented in Table 1. Firstly, IPPmax values were recorded using manual pumping irrigation and a 9.5/11.5 UAS in three different settings. In every measurement, the TFL was used in 1 J-60 W settings. The first setting measured IPPMax while activating the high-power TFL in the center of the pelvis, the second setting included IPPMax measurements while lasing soft stones, and the third setting included measuring IPPMax while lasing hard stones. The mean recorded IPPMax in the center of the pelvis was 18.42 ± 1.83 cmH_2_O. Furthermore, the mean recorded IPPMax when breaking soft stones was 25.83 ± 2.25 cmH_2_O, while the mean IPPmax when treating hard stones was 28.92 ± 1.62 cmH_2_O.

The same measurements were recorded without the use of a UAS. The mean IPPmax when the laser was activated in the center of the pelvis was 55 ± 2.374, while the mean IPPmax while lasing soft and hard stones was 61.83 ± 5.98 and 65.42 ± 5.79, respectively.

**Figure 4:**
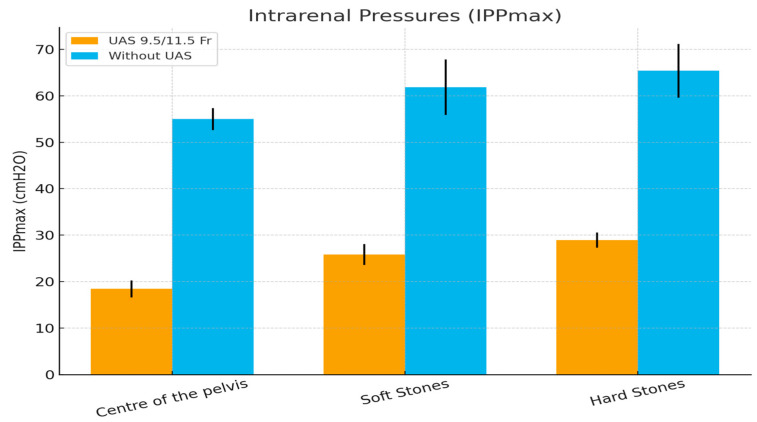
Graph Chart of the outcomes.

The statistical analysis showed that the use of a UAS under pumping irrigation significantly reduced (*p* < 0.05) the IPPMax in all three settings compared to the group where a UAS was not used (Table 1) (Figure 4).

## 4. Discussion

In this study, when a UAS was used, the mean IPPmax in the setting when we activated the laser in the center of the pelvis was measured at less than 20 cm H_2_O. The mean IPPmax, when soft stones were treated, was measured higher than the previous setting but still under the safety limit of 30 cm H_2_O. Furthermore, the mean IPPmax, when hard stones were treated, was measured even higher than the setting with the soft stones (28.92 ± 1.62 vs. 25.83 ± 2.25). However, in all three settings, the mean IPPmax remained under the limit of 30 cmH_2_O. When the UAS was not used, the IPPmax in all measurements exceeded the safe limits. Interestingly, a similar pattern in the increase in the IPPmax has been recognized. Activating the high-power laser in the center of the pelvis resulted in IPPmax as high as 55 cmH_2_O. When treating soft and hard stones, the IPPmax increased even more, exceeding the levels of 65 cmH_2_O in the latter setting. A possible explanation for the increased IPPmax during stone treatment compared to the simple activation of the laser in the center of the pelvis is the increase in IPPmax due to scope manipulation [2,3].

Ureteral access sheaths are a useful tool in everyday urological practice. Their advantages include better control of the irrigation fluids since the fluid is diverted extracorporeally and easier and more direct access of the scope to the area of treatment, which subsequently improves the ability to remove stone fragments and protects the ureteral wall from possible repeated entrances in the ureter [17]. Another significant finding is the reduction in infectious complications when a UAS is used [17]. A number of studies have proved the advantages of using a UAS to retain the IPPMax and the intrarenal temperatures at safe levels. However, the use of a UAS carries well-known risks and could lead to complications such as ureteral wall injuries and ureteral strictures [6,18]. Since the diameter of a normal ureter ranges from 6 to 9 Fr [19], ureteral dilation by inserting a UAS can sometimes be tricky. Pre-stented patients show higher success rates in UAS insertion [20]. A study from Aykanat et al. [21] comparing the use of 9.5/11.5 Fr and 12/14 Fr UAS during RIRS showed that it significantly increases the risk of high-grade ureteral injuries. The same study states that none of the patients were pre-stented. The findings of this study, if replicated in large-scale studies, could be of crucial importance in non-prestented patients with large renal stones. Since the combination of narrow access sheaths and high-power lithotripsy could significantly reduce operating time and ureteral damage [21,22]. A global study from CROES in 2015 [17] showed that, while it reduces infectious complications, the use of a UAS is related to more postoperative complications (*p* = 0.041). The novel single-use 7.5 ureteroscopes offer the advantage of using narrower UASs while reserving enough space in the sheath so that the movement of the scope is unhindered and the irrigation fluid excretion is not compromised. In this in vivo experimental study, we evaluated the effect of the combination of a 9.5/11.5 Fr UAS and a 7.5 Fr scope on the IPPmax while treating artificial stones in the pelvis of anesthetized porcine models. To the best of our knowledge, the in vivo real-time assessment of IPPmax using a 9.5/11.5 Fr and a 7.5 Fr single-use ureteroscope has not been assessed in the current literature.

Phenomena such as pyelotubular backflow and pyelovenous backflow occur in increased intrapelvic pressures. According to Osther et al. [2,3], pyelotubular backflow occurs at pressures of 27–41 cmH_2_O, while pyelovenous backflow occurs at pressures of 41–68 cmH_2_O. Increasing intrapelvic pressure even higher in levels over 80–100 mmHg could even lead to forniceal rupture [23]. The abovementioned phenomena are associated with the ascending pathway of uropathogens during retrograde intrarenal surgery [24]. High intrarenal pressures are associated with postoperative infection and urosepsis [4,25]. According to the findings of our study, it is evident that using a 9.5/11.5 Fr UAS could possibly avoid intrarenal backflow phenomena while using a high-power laser with a 7.5 Fr scope without UAS increases IPPmax to levels susceptible to intrarenal backflow phenomena and thus potentially dangerous for complications. On the contrary, this claim could be supported by the findings of a scientific group studying the histological changes and fluid extravasation using ink in an in vivo study using porcine models [26]. According to their findings, under 100 mmHg pressure, the ink tissue penetration was 0% in the group using a UAS, while it was 31% without a UAS. In the setting of 200 mmhg, the percentage in the UAS group was 18.8% compared to the 99.3% in the group without the UAS. The group concluded that there was a significant difference in the tissue penetration of the kidneys with and without a UAS (*p* = 0.0354). In contrast to the aforementioned data, Yoshida et al. supported that 9.5/11.5 Flexor^@^ UAS was associated with higher IPP [27]. Thus, we may conclude that the use of thinner flexible ureteroscopes may maintain the equilibrium between the IPP and the irrigation outflow.

Noureldin et al. [28] in a similar in vivo experimental study measured IPPmax during ureterorenoscopy with a reusable digital ureteroscope under manual pumping and with different-sized UASs found that IPPmax remained at levels under 30 mmH_2_O only when the 12/14 Fr and the 14/16 Fr ureteral access sheaths were used. Compared to the current study, in similar settings, the use of a 7.5 Fr single-use scope with the 9.5–11.5 Fr UAS seems to prevail compared to the setup with the reusable scope that was used by Noureldin et al. [28]. However, there is an agreement in both studies that the use of a UAS significantly reduces IPPmax in most of the settings. Doizi et al. [29] used a silicone model to evaluate IPP levels during flexible ureteroscopy without using a UAS and with 10/12 Fr, 11/13 Fr, and 12/14 UASs and concluded that the presence of a UAS significantly reduces IPP. Interestingly, in the setup using a 10/12 UAS and a 273 laser fiber in a 9.5 Fr single-use scope, when a UAS was not used, the mean IPP exceeded 30 cmH_2_O, while when a UAS was used with the same setup, the IPP remained under the limit of 30 cmH_2_O. These conclusions are in accordance with the findings of the current study.

The better irrigation outflow favors the safety profile that is offered by the use of a UAS. De Coninck et al. [30] reported a decrease of 57–75% in the transmission of irrigation pressure to the pelvicalyceal system and claimed that using a UAS, IRP remains under 30 cmH_2_O. Furthermore, a study measuring IPP during ureterorenoscopy in human cadaveric kidneys while using 10/12 F, 12/14 F, and 14/16 F UASs and a 7.5 flexible ureteroscope concluded that in every setup, IPP remained under 30 cmH_2_O [31]. These findings are directly comparable with the data from our current study, proving the safety of the 0.78 endoscope-sheath diameter ratio, as Shi et al. have already mentioned [32].

The miniaturization of single-use ureteroscopes has been a breakthrough in modern stone disease treatment. This study shows evidence that using smaller diameter UASs paired with the new single-use scopes and high-power lasers keeps intrapelvic pressures at safe levels. While the beginning of a new era of new technological inventions, such as suctioning ureteral access sheaths and live-measuring ureteroscopes, is on its rise [14,33], acknowledging possible optimal setups without live pressure monitoring that can work under safe conditions could work as a guide to every endourologist, especially in healthcare systems that operate under limited resources.

To our knowledge, this study is one of the few to assess the IPPmax in an in vivo setting of an anesthetized porcine model using a 7.5 Fr single-use scope with and without a 9.5/11.5 Fr UAS. Even with a limited number of porcine models due to Russell’s 3R consideration, that is taken into account, and sample practical considerations such as housing, veterinarian care, and costly interventions that were required, this study opens new horizons for high-power lithotripsy combined with minimized ureteroscopes. The known advantages of high-power lithotripsy could be proven game-changing when treating a large stone burden. Furthermore, this feasibility study supports the safety of using miniaturized scopes and ureteral access sheaths in terms of intrarenal pressure. Considering the great evolution in the laser power field, high-power lithotripsy using scopes of smaller caliber may be considered as a safe approach.

However, several limitations should be noted. Characteristically, the small number of pigs, which is dictated by the limited availability of the animals and possible force variability during the pump irrigation, could affect the fluid irrigation rate. Moreover, the design of this study was created to simulate the operating conditions of a clinical scenario, and thus, the pumped irrigation type was selected, and the gravity irrigation type was excluded since there are studies that prove the possible thermal injury when gravity irrigation is paired with high-power lithotripsy [34]. Similarly, wider ureteral access sheaths were excluded since the main point of the study was to evaluate the safety of using the combination of the 9.5–11.5 Fr UAS and the 7.5 Fr scope, which offers the miniaturization advantages compared to wider UASs. In addition, artificial stones were utilized for this experimental study; however, their similarity with human stones has already been investigated [16]. Lastly, the experimental nature of the study shows some evidence that needs to be validated in large clinical studies.

## 5. Conclusions

Novel 7.5Fr single-use ureteroscopes, when paired with a 9.5 Fr UAS, appear to be a safe and effective combination even in high-power RIRS. While not using a UAS in a similar setup could increase the IPPmax to unsafe levels, that could lead to postoperative complications. Larger clinical studies could strengthen the credibility of these findings, given the experimental animal in vivo nature of the study.

## Figures and Tables

**Table 1:** Maximal intrapelvic pressures (IPPmax) under different settings (mean ± SD).

Intrarenal Pressures IPPmax (cmH_2_O)	UAS 9.5/11.5 Fr	Without UAS	*p*-Value
Center of the pelvis	18.42 ±1.832	55 ± 2.374	*p* = 0.0005
Soft Stones	25.83 ± 2.25	61.83 ± 5.98	*p* = 0.0005
Hard Stones	28.92 ± 1.62	65.42 ± 5.79	*p* = 0.0005

## Data Availability

The data that support the findings of this study are available from the corresponding author upon reasonable request.
